# Prevalence of vascular complications in Ehlers-Danlos syndrome: a systematic review and meta-analysis

**DOI:** 10.1186/s13023-025-03854-6

**Published:** 2025-06-20

**Authors:** Abdelaziz A. Awad, Ambana Yappalparvi, Mahalaqua Nazli Khatib, Roopashree R, Mandeep Kaur, Manish Srivastava, Amit Barwal, G. V. Siva Prasad, Pranchal Rajput, Rukshar Syed, Gajendra Sharma, Anand Prasoon, Muhammed Shabil, Ankit Punia, Megha Jagga, Rachana Mehta, Sanjit Sah, Prakasini Satapathy, Abhay M Gaidhane, Edward Mawejje, Ganesh Bushi

**Affiliations:** 1 Faculty of Medicine, Al-Azhar university, Cairo, Egypt; 2 Noida Institute of Engineering and Technology (Pharmacy Institute), Greater, Noida India; 3 Division of Evidence Synthesis, Global Consortium of Public Health and Research, Datta Meghe Institute of Higher Education, Wardha, India; 4 Department of Chemistry and Biochemistry, School of Sciences, JAIN (Deemed to be University), Bangalore, Karnataka India; 5 Department of Allied Healthcare and Sciences, Vivekananda Global University, Jaipur, Rajasthan, 303012 India; 6 Department of Endocrinology, NIMS University, Jaipur, India; 7 Chandigarh Pharmacy College, Chandigarh Group of College, Mohali - 140307, Jhanjeri, Punjab India; 8 Department of Chemistry, Raghu Engineering College, Visakhapatnam, 531162 Andhra Pradesh India; 9 School of Applied and Life Sciences, Division of Research and Innovation, Uttaranchal University, Dehradun, India; 10 IES Institute of Pharmacy, IES University, Bhopal, 462044 Madhya Pradesh India; 11 New Delhi Institute of Management, Tughlakabad Institutional Area, New Delhi, India; 12 Graphic Era Institute of Medical Sciences, Graphic Era (Deemed to be University, Clement Town, Dehradun, India; 13 Center for Global Health Research, Saveetha Institute of Medical and Technical Sciences, Saveetha Medical College and Hospital, Saveetha University, Chennai, India; 14 Faculty of Data Science and Information Technology, INTI International University, Nilai, Malaysia; 15 Centre of Research Impact and Outcome, Chitkara University, Rajpura, 140417 Punjab India; 16 Chitkara Centre for Research and Development, Chitkara University, Himachal Pradesh, 174103 India; 17 Clinical Microbiology, RDC, Manav Rachna International Institute of Research and Studies, Faridabad, 121004 Haryana India; 18 Department of Paediatrics, Research Centre, Dr. D. Y. Patil Medical College Hospital, Dr. D. Y. Patil Vidyapeeth (Deemed-to-be-University), Pimpri, Pune, 411018 Maharashtra India; 19 Department of Medicine, Korea Universtiy, Seoul, South Korea; 20 University Center for Research and Development, Chandigarh University, Mohali, Punjab India; 21 Medical Laboratories Techniques Department, AL-Mustaqbal University, 51001 Hillah, Babil, Iraq; 22 Global Health Academy, School of Epidemiology and Public Health, Jawaharlal Nehru Medical College, Datta Meghe Institute of Higher Education, Wardha, India; 23 School of Public Health, Makerere University College of Health Sciences, Mulago Hill, Kampala, Uganda; 24 School of Pharmaceutical Sciences, Lovely Professional University, Phagwara, India

**Keywords:** Ehlers-Danlos syndrome, Meta-analysis, Vascular complications, Prevalence

## Abstract

**Background:**

Ehlers-Danlos Syndrome (EDS) comprises connective tissue disorders associated with increased vascular complication risks. This meta-analysis assesses the prevalence of vascular complications in among patients with EDS.

**Methods:**

The review was conducted following PRISMA guidelines. A comprehensive literature search was conducted in PubMed, Embase, and Web of Science until November 2024. Observational studies reporting vascular complications in EDS were included. Data extraction included demographics, complication types, and study design, and quality assessment was evaluated using the modified Newcastle-Ottawa Scale (NOS). Random-effects models and I² statistics assessed heterogeneity, while Doi plots evaluated publication bias.

**Results:**

Of the 1,772 articles screened, 12 met the inclusion criteria, reporting various vascular complications in EDS. The overall pooled prevalence of vascular complications was 30.03% (95% CI: 15.00–51.07%). The prevalence for the vEDS subtype was 42.36% (95% CI: 12.63–78.88%), for unspecified EDS was 18.65% (95% CI: 5.38–48.03%), and for hEDS was 19.77% (95% CI: 15.09–25.16%). Sensitivity analyses confirmed the stability of the pooled prevalence estimates, and DOI plots indicated minimal publication bias.

**Conclusions:**

This review highlights the high risk of vascular complications in vEDS, with moderate involvement in other EDS subtypes. Regular vascular monitoring, especially in vEDS, is crucial for early detection and intervention. Standardized diagnostic protocols and further research into genetic factors are needed to improve management strategies.

**Supplementary Information:**

The online version contains supplementary material available at 10.1186/s13023-025-03854-6.

## Introduction

Ehlers-Danlos Syndrome (EDS) encompasses a group of inherited connective tissue disorders characterized by symptoms such as stretchy skin, joint looseness, and tissue fragility [1–3]. The vascular subtype (vEDS) is the most severe, presenting a high risk of sudden, serious complications like arterial tears, due to mutations in the COL3A1 gene that weaken blood vessel walls [4–6]. Each EDS subtype has unique features: classical EDS (cEDS) involves stretchy, bruise-prone skin linked to COL5A1 or COL5A2 mutations; hypermobility EDS (hEDS), the most common form, is characterized by joint looseness and chronic pain but lacks a specific genetic marker [7–9]. Other types include kyphoscoliotic EDS (kEDS), which causes severe muscle weakness due to PLOD1 mutations, arthrochalasia EDS (aEDS) with extreme joint laxity resulting from COL1A1 or COL1A2 mutations, and dermatosparaxis EDS (dEDS) with fragile skin due to ADAMTS2 mutations [10–12]. Rare types such as myopathic, spondylodysplastic, and periodontal EDS further add to the clinical complexity [13–15].

In vEDS, defective collagen synthesis impairs blood vessel structure, particularly in medium and large arteries, making them susceptible to rupture, dissection, and aneurysm formation [16–18]. These vascular complications are frequent and severe in vEDS, distinguishing it from other EDS types where such events are less common [19–21]. Understanding the frequency of these complications is essential for effective clinical management, as these events are often sudden and life-threatening. However, despite the clinical importance of these complications, data on their frequency remains inconsistent across studies, partly due to differences in research designs and diagnostic criteria [22–24].

This systematic review and meta-analysis aimed to quantify the prevalence of vascular complications across various subtypes of EDS, providing a comprehensive overview of the risks associated with this group of disorders. By synthesizing data from existing studies, this review offered valuable insights into the incidence of vascular events and helped inform clinical practices and guidelines for managing EDS patients.

## Method

This meta-analysis followed PRISMA guidelines [25] (Table S1), and the study protocol was registered with the PROSPERO (CRD42024608932) database.

### Eligibility criteria

The study included observational research designs, specifically cohort studies (both prospective and retrospective), case-control studies, and cross-sectional studies, that reported quantitative data on the prevalence of vascular complications among patients diagnosed with EDS. Case reports, narrative reviews, editorials, and studies lacking a clear emphasis on EDS or comprehensive data on vascular complications were excluded. Only studies published in English were included to ensure consistency in interpretation, as no translation services were utilized. Additionally, there were no explicit geographical location restrictions; studies from all regions were considered eligible, provided they met the inclusion criteria (Table S2).

### Search strategy

We performed a literature search in PubMed, Embase, and Web of Science databases up to November, 2024. The search utilized a combination of keywords and MeSH terms, including “Ehlers-Danlos Syndrome,” “Ehlers Danlos Disease,” “Elastic Skin Syndrome,” “Rubber Man Syndrome,” combined with terms like “Microangiopathy,” “Vascular incident,” “vascular EDS,” “vascular complications,” and “Vascular impairment.” Boolean operators (AND, OR) were applied to refine and broaden the search for relevant studies (Table S3).

### Screening and data extraction

The screening and data extraction process was conducted using Nested Knowledge software. In the first stage, titles and abstracts of identified studies were reviewed to exclude articles that did not meet the predefined eligibility criteria. In the second stage, full-text reviews were conducted for studies that appeared to meet the inclusion criteria. Screening at both stages was conducted independently by two reviewers, with any disagreements resolved through consultation with a third reviewer to ensure consistency and reduce bias.

Data extraction was performed using the tagging function in Nested Knowledge. Key information collected included study characteristics (author, publication year, geographic location, study design, and sample size) and detailed information on vascular complications (types and reported events). Data extraction was independently performed by two reviewers to enhance accuracy and minimize errors. Any discrepancies in the extracted data were addressed through discussion and consensus.

### Quality assessment

The quality of the included studies was evaluated using the Modified Newcastle-Ottawa Scale (NOS), a tool designed to assess the methodological rigor of non-randomized studies. Points were assigned to each study based on adherence to specific criteria, with total scores ranging from 0 to 6. Studies were categorized into three quality levels: high quality (5–6 points), moderate quality (3–4 points), and low quality (0–2 points). This scoring system provided a standardized approach to evaluate the reliability and validity of the included studies [26].

### Statistical analysis

Meta-analysis was conducted using R software version 4.4, applying random-effects models to account for variability among studies [27, 28]. Heterogeneity was assessed using the I² statistic to quantify variation due to differences across studies [29]. Subgroup analyses were performed to examine prevalence variations among EDS subtypes. Publication bias was evaluated using Doi plots and LFK index values to detect asymmetry in effect size distribution. Sensitivity analysis was conducted with a leave-one-out approach to assess the influence of individual studies on the overall pooled results, ensuring robustness and stability of the findings [30, 31].

## Results

**Fig. 1:**
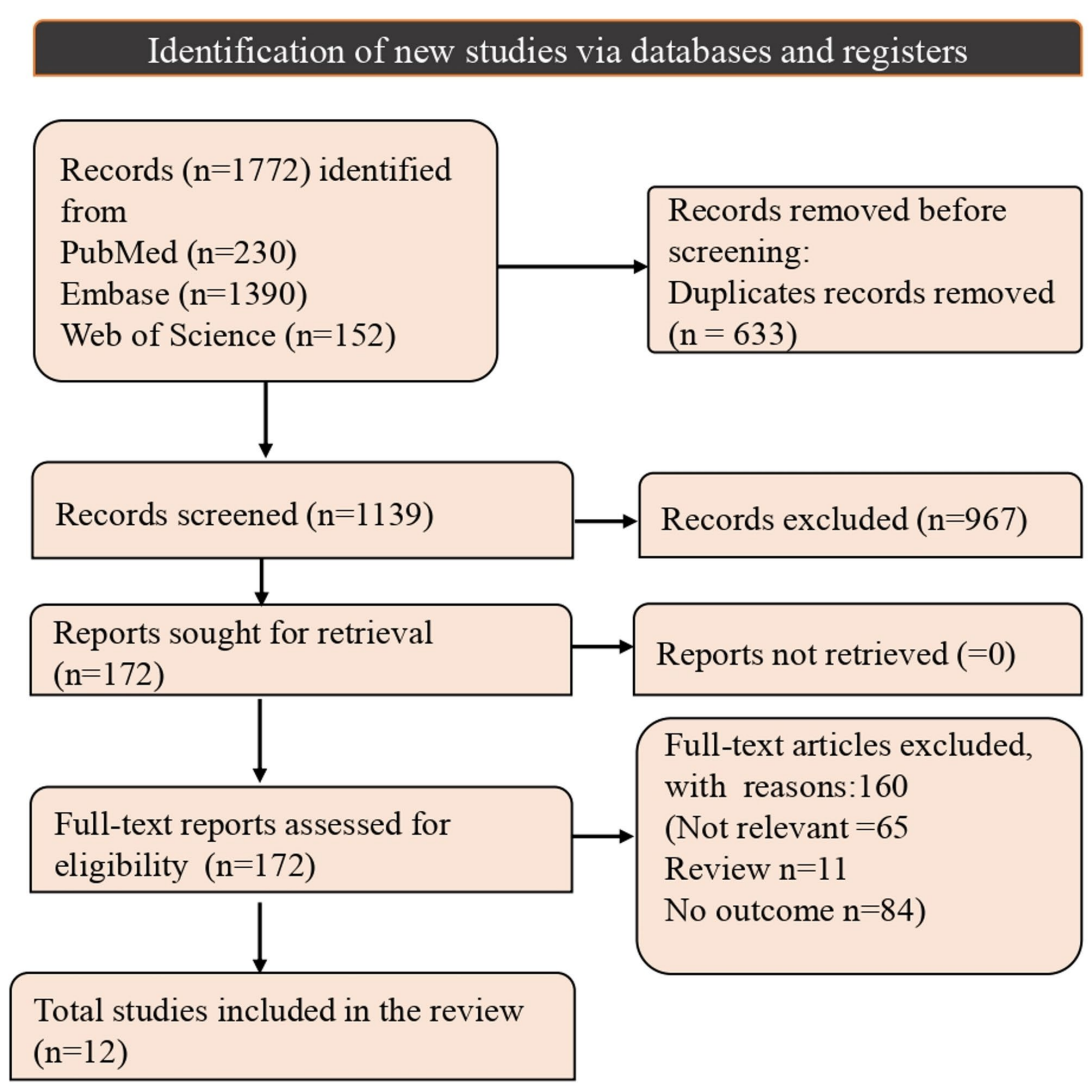
PRISMA flowchart showing the selection process of included studies

A total of 1,772 records were identified through database searches, including 230 from PubMed, 1,390 from Embase, and 152 from Web of Science. After the removal of 633 duplicates, 1,139 records remained for screening. Titles and abstracts were reviewed against predefined eligibility criteria, resulting in the exclusion of 967 records. Subsequently, 172 articles underwent full-text screening to assess their relevance and outcomes. Of these, 160 articles were excluded, including 65 due to irrelevance. Ultimately, 12 studies met the inclusion criteria and were included in the meta-analysis (Fig. 1).

### Summary of studies investigating vascular complications in EDS

Twelve studies on vascular complications in EDS were included. Sample sizes ranged from 27 to 476 participants, with female representation varying between 50% and 89%, and ages ranging from 1 to 73 years. The included studies comprised eight retrospective cohort studies, three cross-sectional studies, and one multicenter retrospective study. Reported vascular complications included sudden arterial lesions, medium-sized artery complications, aortic lesions, aneurysms, abdominal complications, coronary artery disease, peripheral vascular disease, neurovascular complications, vascular fragility, carotid-cavernous fistula, cardiovascular complications, mild aortic root and ascending aorta dilation, and complications of the iliac, hepatic, celiac, renal, and splenic arteries, as well as mitral regurgitation, thoracic aortic dilation, and arterial aneurysms (Table 1). The quality of the studies, assessed using the modified NOS, was determined to be moderate to high (Table S4).

**Table 1:** Summary characteristics of studies investigating vascular complications in EDS

Author	Study design	Population	Female (%)	Ages (Year)	No. of patients with EDS	Prevalence of vascular complication (%)	Summary of vascular complications
Adham_2021 [52]	Retrospective Cohort Study	vEDS	NA	35 (mean)	144	Sudden Arterial Ears Lesions = 56.94%	Sudden arterial lesions were frequent, indicating severe arterial fragility in vEDS patients.
Adham_2022 [53]	Retrospective Multi Centric Cohort Study	vEDS	NA	36 (mean)	330	Medium Sized Arteries = 82.42% Aortic Lesions Alone = 2.72% MSA And Aortic Lesions = 10.90%	High prevalence of medium-sized artery complications and combined vascular events, underscoring systemic vascular fragility in vEDS.
Demirdas_2024 [54]	Retrospective Study	vEDS	50%	15–61	142	Aneurysm outside aorta = 12.6% Dissection outside aorta = 13.38% Abdominal complications = 6.33%	Diverse vascular complications, including significant aneurysms and dissections outside the aorta, highlight systemic vascular risks.
Ghoraba_2023 [55]	Retrospective Study	Unspecified subtype EDS	87%	40 (mean)	307	Retinal Arterioles = 84.69% Macular Arterioles = 35.50%	High prevalence of retinal and macular arteriole complications suggests vascular involvement in ocular systems in unspecified EDS.
Jayarajan_2020 [56]	Retrospective Study	Unspecified subtype EDS	NA	55 (mean)	476	Coronary Artery Disease = 12.51 Peripheral Vascular Disease = 8.61%	Coronary and peripheral vascular complications indicate systemic vascular challenges beyond arterial fragility.
Nourissat_2018 [57]	Retrospective Study	Unspecified subtype EDS	70%	< 14	27	29.62%	Neurovascular complications highlight the broad spectrum of vascular risks even in younger patients with unspecified EDS.
Oderich_2005 [58]	Retrospective Study	vEDS	55%	28 (mean)	29	Vascular Fragility = 89.65%	High prevalence of carotid-cavernous fistulas and vascular fragility underscores severe risks in vEDS.
Paige_2019 [59]	Retrospective Study	Unspecified subtype EDS	76%	1–60	95	Cardiovascular complication = 11.57%	Cardiovascular complications were noted, though with lower prevalence compared to other vascular events in unspecified EDS.
Pietri-Toro_2023 [60]	Retrospective Study	hEDS	NA	NA	75	Mild Aortic Root Dilation = 1.33% 1 Mild Ascending Aorta = 1.33%	Mild aortic involvement was observed, reflecting relatively limited vascular complications in hEDS.
Shalhub_2019 [61]	Cross-Sectional Cohort Study	vEDS	NA	41 (mean)	33	Iliac Artery = 69.69% Artery Complications = 24.24% Celiac Artery Complications = 42.42% Renal and Carotid Artery Complications = 51.51% Splenic Artery Complications = 33.33%	Multiple vascular complications in medium and large arteries illustrate widespread vascular fragility in vEDS patients.
Stephens_2020 [62]	Retrospective Study	hEDS	89%	4–73	258	Mitral Regurgitation = 6.20% Thoracic Aortic Dilatation = 19.76% Aortic Dilatation = 7.36%	Cardiac involvement, including mitral regurgitation and thoracic aortic dilation, was noted in hEDS with moderate prevalence.
Wang_2021 [63]	Retrospective Study	vEDS	58%	38 (mean)	68	Arterial Aneurysms =57.35	High prevalence of arterial aneurysms highlights the significant vascular risks associated with vEDS.

Abbreviations: vEDS– Vascular Ehlers-Danlos Syndrome, hEDS– Hypermobility Ehlers-Danlos Syndrome, cEDS– Classical Ehlers-Danlos Syndrome, NA– Not Available, MSA– Medium-Sized Arteries

### Meta-analysis

#### Prevalence of vascular complications in EDS

**Fig. 2:**
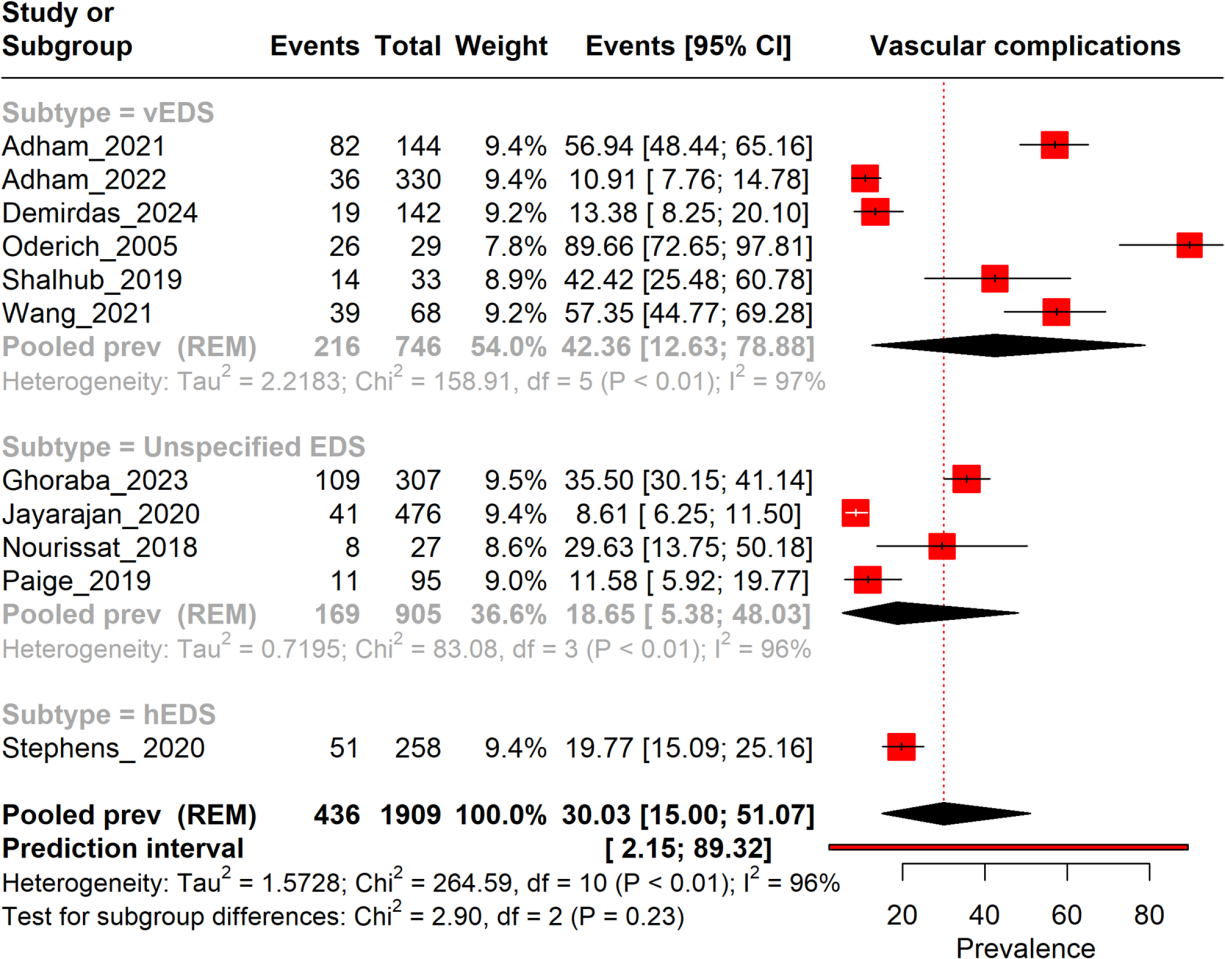
Forest plot presenting the prevalence of vascular complications in EDS

The overall pooled prevalence of vascular complications in EDS was 30.03% (95% CI: 15.00–51.07%), with a wide prediction interval of 2.15–89.32%, reflecting substantial variability across studies. Among subtypes, vEDS showed the prevalence of 42.36% (95% CI: 12.63–78.88%), consistent with its known severity and vascular fragility. Unspecified EDS demonstrated a prevalence of 18.65% (95% CI: 5.38–48.03%), likely influenced by varied diagnostic criteria, while hEDS had a prevalence of 19.77% (95% CI: 15.09–25.16%), aligning with its milder clinical phenotype. High heterogeneity (I² > 95%) across all subtypes underscores variability in study populations, methodologies, and diagnostic definitions (Fig. 2).

### Sensitivity analysis

**Fig. 3:**
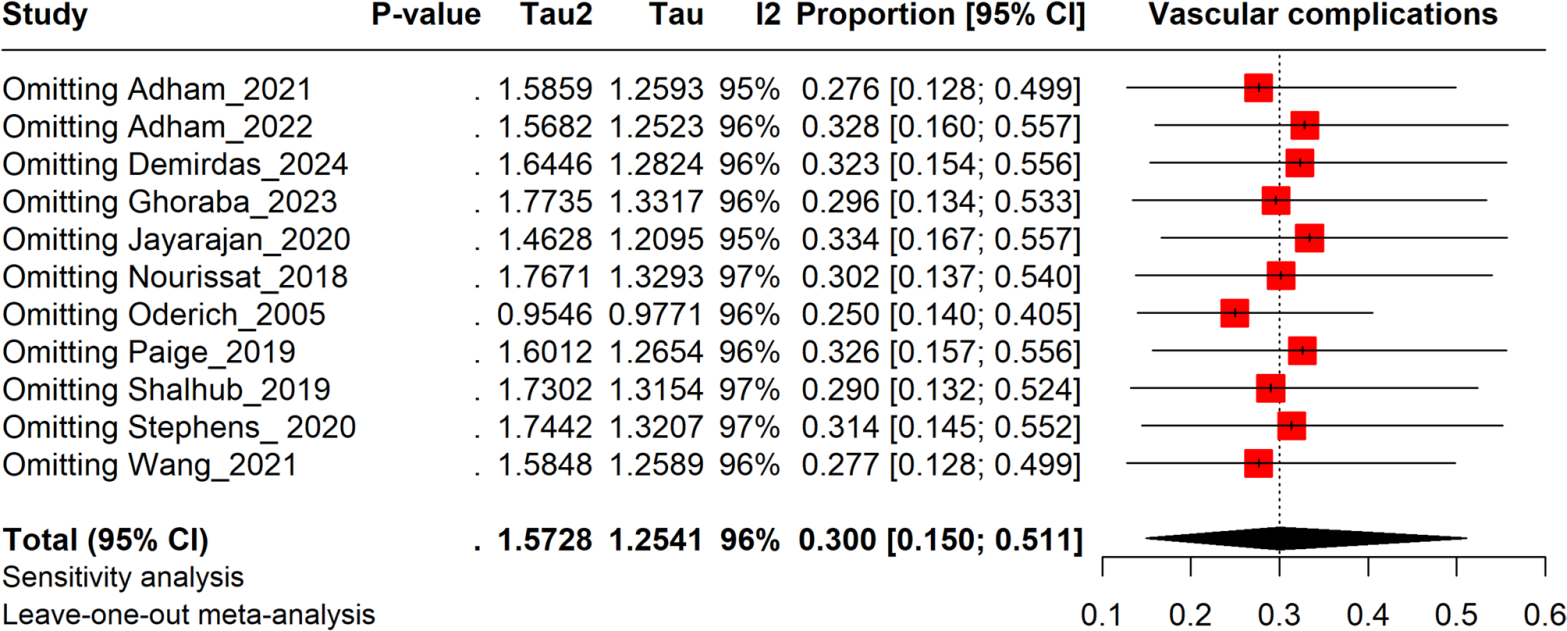
Sensitivity analysis plot illustrating vascular complications in EDS

The leave-one-out sensitivity analysis confirms the stability of the pooled prevalence of vascular complications in EDS. The recalculated prevalence ranges from 25.0% (95% CI: 14.0–40.5%) to 33.4% (95% CI: 16.7–55.7%), with the overall pooled prevalence at 30.0% (95% CI: 15.0–51.1%). High heterogeneity (I²: 95–97%) persists, reflecting variability across studies that is not attributable to any single study (Fig. 3).

### Publication bias

**Fig. 4:**
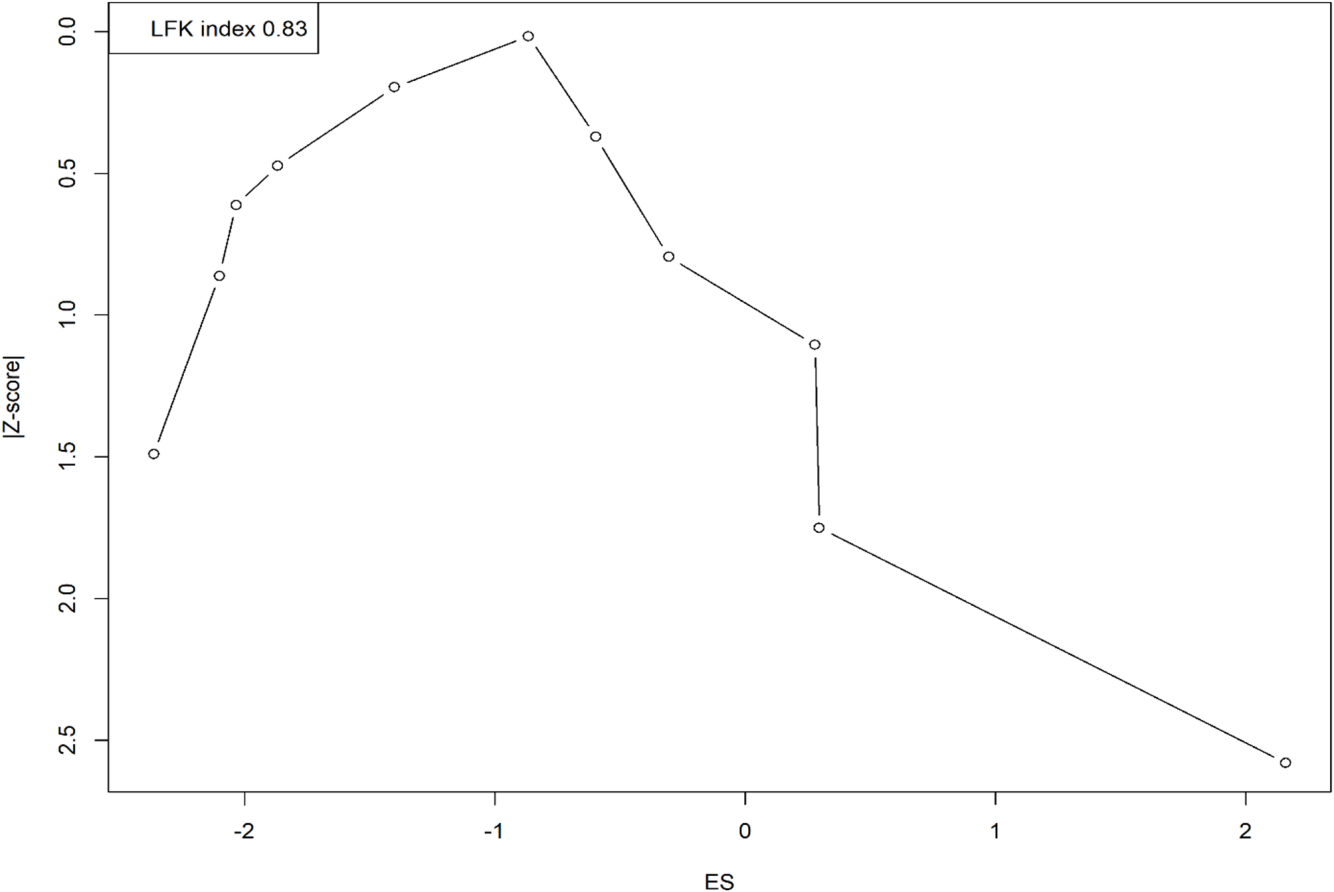
Doi plot assessing publication bias in studies on vascular complications in EDS

The DOI plot, with an LFK index of 0.83, shows no major asymmetry, indicating minimal evidence of publication bias (Fig. 4).

## Discussion

The prevalence of vascular complications in EDS, particularly in vEDS, is notable and underscores the need for targeted management. The meta-analysis demonstrated variation in vascular complication rates among subtypes. The prevalence in vEDS was 42.36%, reflecting its severe vascular fragility and increased risk of arterial rupture. In unspecified EDS, the prevalence was 18.65%, likely influenced by inconsistent diagnostic criteria, while hEDS exhibited a prevalence of 19.77%, consistent with its less severe vascular involvement. High heterogeneity (I² > 95%) indicates substantial variability in study populations and methodologies, highlighting the need for further research to refine prevalence estimates. Genetic predispositions and systemic factors may influence this variability, as studies have shown that genetic variability and adipocytokines play roles in vascular risks in EDS. Additionally, genetic factors have been reported to significantly contribute to these risks, either by offering protection or increasing susceptibility to complications [32–34].

Recent research has expanded the understanding of the genetic and clinical landscape of EDS, particularly its vascular complications. Ochoa Chaar et al. emphasized the role of genetic predispositions in the pathogenesis of peripheral arterial disease, underscoring the broader relevance of genetic evaluation in connective tissue disorders such as EDS [35]. Similarly, Sedky et al. highlighted systemic manifestations like obstructive sleep apnea in patients with EDS and Marfan syndrome, illustrating the multi-organ burden of heritable connective tissue disorders [36]. Although the present meta-analysis included 12 studies, additional evidence illustrates the substantial and diverse vascular risk associated with EDS, especially in vEDS.

In a national cohort of 142 patients with genetically confirmed vEDS in the Netherlands, 48% experienced arterial aneurysms or dissections, with a median age at first vascular event of 44 years, and 41% had multiple vascular events, underscoring the recurrent and progressive nature of vascular fragility [37]. Furthermore, a systematic review involving 448 patients across various EDS subtypes reported 720 aneurysms, primarily in the abdominopelvic (*n* = 386) and intracranial (*n* = 165) regions, suggesting diffuse vascular involvement beyond traditional anatomical boundaries [38]. Cerebrovascular complications also appear disproportionately elevated in this population. A hospitalization-based study revealed markedly increased odds of carotid artery dissection (odds ratio [OR], 15.02) and cerebral aneurysm (OR, 5.59) in EDS patients compared with matched controls [39].These findings highlight the increased risk of life-threatening neurovascular events and support the need for proactive vascular surveillance, particularly in genetically confirmed vEDS.

The mechanisms underlying vascular complications in vEDS are closely linked to mutations in the **COL3A1** gene, which encodes type III collagen, a critical structural component of the arterial wall [40]. Mutations in this gene compromise collagen fibril stability, leading to reduced vessel wall strength and a marked increase in the risk of rupture, aneurysm, and dissection hallmarks of vEDS [41]. The pathogenicity of these mutations is further exacerbated by dysregulated transforming growth factor-beta (TGF-β) signaling, which promotes extracellular matrix remodeling and contributes to vascular fragility [42–44]. This genetic basis highlights the necessity for genotype-informed monitoring and management strategies [45–47].

Despite these insights, the relationship between genotype and phenotype in vEDS remains complex and incompletely understood. Mutations in **COL3A1** can lead to a spectrum of clinical outcomes, ranging from early-onset, life-threatening vascular events to milder manifestations [48, 49]. This heterogeneity suggests that additional genetic modifiers, such as polymorphisms in collagen-related pathways and epigenetic factors, may influence phenotypic expression [50, 51]. Understanding these genotype-phenotype relationships is essential to improve risk stratification, early diagnosis, and personalized management of vascular complications in EDS.

This systematic review provides a comprehensive assessment of the prevalence of vascular complications across EDS subtypes using a robust methodology, including systematic searches of PubMed, EMBASE, and Web of Science, along with quality appraisal via the Modified Newcastle-Ottawa Scale. However, several limitations should be acknowledged. The high degree of heterogeneity likely attributable to differences in study design, diagnostic criteria, and reporting practices complicates precise prevalence estimation. The predominance of retrospective studies may result in underreporting of milder or atypical vascular events. Moreover, publication bias where studies with significant or novel findings are more likely to be published could lead to overestimation of complication rates. Although funnel plots and Egger’s test were applied to assess this bias, the limited number of studies restricts the reliability of these assessments.

Furthermore, exclusion of case reports and small case series often valuable sources of data in rare diseases may have narrowed the evidence base. Notably, the literature beyond the included studies describes rare presentations such as spontaneous visceral artery rupture in classical EDS and cerebral aneurysms in kyphoscoliotic EDS, highlighting the need for inclusive data synthesis. Additionally, restricting the review to English-language publications may have excluded relevant findings from non-English studies. These limitations underscore the need for future research efforts, including disease registries and prospective cohort studies that incorporate broader EDS populations, standardized vascular phenotyping, genetic confirmation, and long-term follow-up. Such initiatives would improve the precision of prevalence estimates and foster a more nuanced understanding of vascular burden across the EDS spectrum.

The findings of this meta-analysis underscore the importance of routine vascular surveillance in patients with EDS, especially those with vEDS. Clinicians should consider implementing regular imaging and cardiovascular assessments to detect complications early and enable timely interventions. Early identification and management of vascular complications may improve clinical outcomes and quality of life in this high-risk population.

From a policy perspective, this study highlights the necessity for standardized diagnostic criteria and consistent reporting protocols for vascular events in EDS. Establishing universal standards would reduce heterogeneity across studies, improve the reliability of pooled prevalence estimates, and facilitate more accurate risk stratification. Such consistency would not only benefit clinical practice but also enhance the validity of future research.

Future research should prioritize large, multicenter prospective studies with standardized diagnostic frameworks and rigorous methodologies. These studies are essential to delineate vascular risk profiles across EDS subtypes more accurately. Additionally, further investigation into the genetic and molecular mechanisms underlying these complications will be critical for developing targeted therapies. Understanding gene-environment interactions and genetic modifiers will facilitate personalized approaches to care. Although current evidence regarding sex differences in vascular risk remains inconclusive, future studies should also explore potential gender-related variations. Ultimately, a more refined understanding of the interplay between genetic, molecular, and clinical factors will be key to improving patient outcomes and reducing the healthcare burden associated with vascular complications in EDS.

## Conclusion

This systematic review and meta-analysis underscore the significant risk of vascular events in vEDS, reflecting the severity of vascular fragility inherent to this condition. Other EDS subtypes, such as hEDS, demonstrated moderate vascular involvement. These findings emphasize the importance of regular vascular monitoring in individuals with EDS, particularly in vEDS, to facilitate early detection and intervention. Further research with standardized diagnostic protocols is needed to improve understanding of the genetic factors contributing to vascular complications in EDS, enabling more targeted management strategies.

## Electronic supplementary material

Below is the link to the electronic supplementary material.

Supplementary Material 1: Table S1. PRISMA Checklist. Table S2. Inclusion and Exclusion criteria. Table S3. The adjusted search terms as per searched electronic databases. Table S4. Modified Newcastle-Ottawa Scale (NOS).

## Data Availability

All data generated or analyzed during this study are included in this published article (and its Supplementary information files).
